# A Case Report of Coronary-Subclavian Steal Syndrome Treated with Carotid to Axillary Artery Bypass

**DOI:** 10.1155/2009/687982

**Published:** 2009-07-20

**Authors:** Wissam Al-Jundi, Aiman Saleh, Kathryn Lawrence, Sohail Choksy

**Affiliations:** Colchester Hospital University NHS Foundation Trust, Colchester, Essex CO4 5JL, UK

## Abstract

Coronary-subclavian steal syndrome results from atherosclerotic disease of the proximal subclavian artery causing reversal of flow in an internal mammary artery used as conduit for coronary artery bypass. This rare complication of cardiac revascularisation leads to recurrence of myocardial ischaemia. When feasible, subclavian angioplasty and/or stent placement can provide acceptable result for these patients. Vascular reconstruction through carotid to subclavian artery bypass has been the standard procedure of choice. Other interventions in literature include axilloaxillary bypass and subclavian carotid transposition. This case report describes the use of carotid axillary artery bypass for the treatment of coronary-subclavian steal syndrome.

## 1. Introduction

The use of left internal mammary artery (LIMA) is recommended as a coronary bypass graft due to its excellent long-term results [1]. However, the development of proximal subclavian stenosis may lead to reversal of flow from the LIMA to subclavian artery resulting in coronary artery disease known as coronary-subclavian steal syndrome (CSSS).

## 2. Case Report

A 73-year-old man was admitted with severe chest pain at rest for 3 hours. He had previous three myocardial infarctions with the last one occurring 14 years before this admission. At that time, he underwent coronary artery bypass grafting (CABG) with LIMA to left anterior descending artery and two saphenous vein grafts to the right coronary and first obtuse marginal arteries. Following surgery, his chest pain resolved, but he had frequent admissions with chest pain over the last four years which was misdiagnosed as coronary artery spasm. In addition, his past medical history included congestive heart failure and cardiac arrest twelve years ago. His coronary risk factors were hypertension, hypercholesterolemia, family history of ischaemic heart disease, and a 45 pack-year history of cigarette smoking.

His physical examination revealed a blood pressure of 124/63 mmHg in the right arm and 83/50 mmHg in the left, a regular pulse of 84 beats/minute, and respiratory rate of 18 breaths/minute. He had normal heart sounds and the lung fields were clear. The vascular examination revealed easily palpable right upper extremity and bilateral lower extremity pulses. Left upper extremity pulses were present but diminished. Carotid arteries were palpable with no audible bruits. No abdominal masses were palpated.

A 12-lead electrocardiogram demonstrated ischaemic changes in lateral leads and a chest x-ray showed cardiomegaly. Cardiac enzymes revealed troponin of 0.05 *μ*g/L. On diagnostic coronary angiography, there was 50% stenosis in left main stem and 50% stenosis in left anterior descending artery in mid-segment with good run off. Failure to pass the catheter through the left subclavian artery indicated possible stenosis raising suspicion of CSSS.

**Figure 1:**
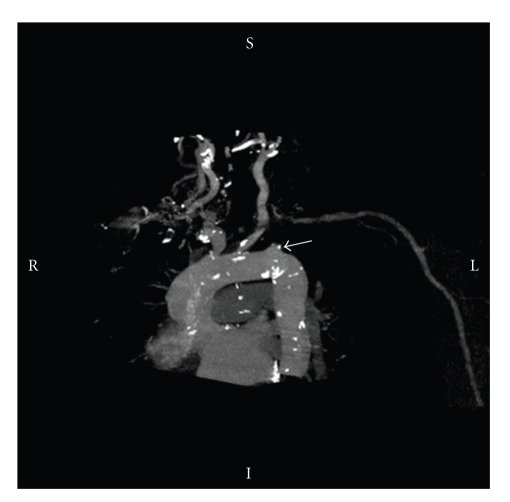
CT angiogram demonstrating the proximal stenosis of the left subclavian artery (arrow).

CT Angiogram confirmed a 2 cm stenosis at the origin of left subclavian artery (Figure 1). Stenting of left subclavian artery was considered hazardous due to the risk of occlusion of the adjacent vertebral artery. The patient was referred to undergo left common carotid to subclavian artery bypass. After exploration, the left subclavian artery was found to have significant atherosclerosis. Therefore, an infraclavicular incision was utilised for access to the left axillary artery. A 6 mm ringed Vascutek PTFE graft (Sulzer Vascutek Ltd.; Renfrewshire, Scotland, UK) was used to construct a bypass between the left common carotid artery and the left axillary artery tunnelled beneath the clavicle. Carotid shunt was not used while systemic heparin (5000 units) was administered during the operation. Postoperatively, a good graft pulse was felt in addition to easily palpable left upper extremity pulses. The patient made an uneventful recovery, the angina pain disappeared and he was discharged 4 days following the operation. Two months later, he reported improvement in symptoms and his blood pressure was equal in both arms. Control CT angiogram nine months later revealed an intact PTFE graft between carotid and axillary artery.

## 3. Discussion

First reported by Harjola and Valle in 1974 [2], CSSS is an uncommon complication of coronary artery bypass using LIMA with an incidence varying between 0.07–3.4% in those requiring coronary grafts [3, 4]. It is most commonly caused by atherosclerotic stenosis of the left subclavian artery proximal to the origin of the in situ LIMA graft leading to myocardial ischemia due to reversal of flow in the coronary conduit [5].

Although occasionally asymptomatic [6], CSSS usually presents as recurrent angina after stress to the upper limb [7] but can also manifest with silent ischemia [8, 9], myocardial infarction [10, 11], or heart failure [12, 13]. Symptoms have been reported to occur between 2–31 years following surgery. Symptoms presenting within a year of CABG usually suggest a subclavian stenotic lesion missed at surgery [14].

Proximal aortic arch arteriography is the gold standard for diagnosing subclavian stenosis or occlusion. Alternative diagnostic procedures are Doppler, duplex ultrasonography, or magnetic resonance angiography [13].

There are several options for treating CSSS. These include surgical and radiological guided endovascular procedures. If there is subclavian artery stenosis in the preoperative evaluation, coronary surgery can be combined with a direct subclavian artery bypass. However, if subclavian artery stenosis is diagnosed after the coronary bypass surgery, the percutanous approach is more recommended due to its less invasiveness, lower complication rates, and shorter hospital stay compared to surgical treatment [15].

Radiological procedures include percutaneous transluminal balloon angioplasty (PTA) and stenting. The advantages of radiological procedures are minimal invasiveness, avoidance of general anaesthesia, less morbidity, and mortality along with excellent short-term results [16]. However, passing a guide wire may be difficult in chronic occlusion and the restenosis rate for angioplasty is reported to be as high as 40.7% over 5 years in patients with CSSS [17, 18], whilst the rate of recurrent stenosis following stenting is about 16% [19, 20].

In our case the patient developed symptoms more than 10 years after CABG. Since the stenosis was very close to the vertebral artery, there was a risk of occlusion during stenting. Therefore, bypass grafting was chosen in preference.

Carotid subclavian artery bypass is considered the standard surgical treatment for symptomatic occlusion of proximal subclavian artery [21]. However, preparing an atherosclerotic subclavian artery in our patient for anastomosis was not feasible. In addition, exposure of the subclavian artery and bypass grafting to it through supraclavicular approach is not without technical difficulty, especially with the close proximity to important lymphatic channels and nerves. On the other hand, Infraclavicular exposure of the axillary artery is a straightforward technique routinely performed by vascular surgeons and eliminates some of the potential risks of supraclavicular approach. A review of 10-year experience with carotid-axillary artery bypass revealed a graft patency rate of 96% over a mean follow up of 47 months [22].

## 4. Conclusion

Coronary-subclavian steal syndrome should be considered in patients presenting with recurrent chest pains after CABG with in situ left internal mammary grafts. Surgical extra anatomical bypass is an effective method of treating this uncommon condition when PTA is not possible. In experienced hands, carotid subclavian artery bypass remains the standard surgical procedure of choice. Carotid axillary artery bypass can be a feasible alternative when the traditional procedures are technically difficult.
